# London University Course

**Published:** 1921-08-27

**Authors:** 


### LONDON UNIVERSITY COURSE.

The University of London confers the degrees of Bachelor of Medicine and Bachelor of Surgery (M.B., B.S.), Doctor of Medicine (M.D.), and Master of Surgery (M.S.).

A student who desires to take the degrees of M.B., B.S. of the University of London must be registered as a matriculated student either by passing the Matriculation examination in one of its forms or by being exempted from the examination on the ground of having passed an *approved* examination of equal standard in another University.

The course in medicine normally extends over five and a-half years from matriculation, and the whole course may, and the greater part must, be taken at one or more of the medical institutions recognised by the University. It is not necessary for a student to apply for registration as a medical student by the General Medical Council in order to qualify for the examinations in medicine of the University.

In the first year of the medical course inorganic chemistry, physics, and general biology in preparation for the first examination for medical degrees are taken, the examination being held in July and December. Organic and applied chemistry is studied in the first half of the second year for Part I. of the second examination, which is taken normally six months after passing the first examination. Part I. of the second examination is held in March and July. The second and third years are devoted to organic and applied chemistry, human anatomy and embryology, physiology, pharmacology, including pharmacy and materia medica. The second medical examination, Part II., is normally taken two and a-half years after matriculation, the examinations being held in March and July.

In the final course for the M.B., B.S. (the two degrees must be taken together), the subjects are medicine, surgery, midwifery and diseases of women, pathology, forensic medicine, and hygiene. The examination may be taken in two groups, and is held in May and October. The examination is written, clinical, oral, and practical. The candidate is required to examine and report on selected cases, to do practical pathological work, and to be acquainted with the essentials of operative surgery, though actual operations on the dead subject are not demanded.

Exemption is granted in certain subjects at the first and second medical examinations to students who have passed other examinations of the University in the same subjects.

The detailed regulations in the Faculty of Medicine for internal students may be obtained from the Academic Registrar, University of London, South Kensington, S.W. 7.

